# 

**Published:** 1883-04

**Authors:** 


					﻿PUBLISHER’S NOTICE.
An unavoidable delay lias occurred in the issue of this the April
number of The Independent Practitioner, the nature of which will be
explained in the May number, when further important announcements
will be made.	_____________________
Contributors:
Alf. L. Loomis, M.D.................New	York
Fessenden N. Otis, M.D.............
F. H. Bosworth, M.D.................. “
J. L. Little, M.D................ .	“
J. Williston Wright, M.D............. “
C. E. Francis, D.D.S., M.D.S......... “
A. J. C. Skene, M.D..........Brooklyn,	N. Y.
O.	A. Marvtn, D.D.S..........
Edwin T Darby, M.D., D.D.S.......Phila.,	Pa.
P.	S. Wales, M.D......Surg.-Gen’l	U. S. Navy
H. F. Campbell, M.D.............Augusta,	Ga.
C. H. Mastin, M.D., LL.D.........Mobile, Ala.
J. J. Chisolm, M. D...........Baltimore, Md.
C. F. Bevan, M.D.................. “
I.	E. Atkinson, M.D............... “
H. McGuire, M.D................Richmond,	Va.
F. P. Porcher, M.D..........Charleston,	S. 0.
Jas. T. Whittaker, M.D........Cincinnati,	O.
J.	Ransohoff, M.D., F.R.C.S....	“
F. Forchheimer, M.D................ “
A. R. Jackson, M.D..............Chicago,	Ill.
S. V. Clevenger, M.D............... “
E. F. Ingals, M.D.................. “
I J. H. Carstens, M.D..........Detroit,	Mich.
PROSPECTUS
Fob Vol. IV., 1883, of the Independent
Pbactitioneb.
This Jottknal is independent of colleges,
cliques, isms or any advertising firm; there-
fore impartiality pervades its columns in the
sole interest of the Profession.
It is cosmopolitan in its circulation and
scientific resources; consequently of equal
interest to the specialist and general prac-
titioner everywhere.
That which is new and useful in Medicine,
Surgery, Obstetrics, Dentistry, Pathology
and Popular Science is given to its readers
in an intelligent and concise style.
				

